# Longitudinal Associations Between Problematic Internet Use and Future Time Perspective Among Adolescents: A Two-Wave Cross-Lagged Study

**DOI:** 10.3390/bs16081412

**Published:** 2026-08-18

**Authors:** Haiping Hao, Erxing He, Geyang Hu, Qiao Hu

**Affiliations:** 1 School of Education, Huainan Normal University, Huainan 232038, China; haohaiping66@hnnu.edu.cn (H.H.); heerxing119@hnnu.edu.cn (E.H.); 2 Time Psychology Research Center, Faculty of Psychology, Southwest University, Chongqing 400715, China; 3 Faculty of Education, University of Macau, Avenida da Universidade, Taipa, Macau 999078, China; mc54179@um.edu.mo

**Keywords:** future time perspective, problematic Internet use, adolescents, longitudinal study

## Abstract

Previous studies have identified associations between problematic Internet use (PIU) and future time perspective (FTP), but their temporal ordering remains unclear. This study examined longitudinal associations between PIU and three FTP dimensions—future-positive, future-negative, and task orientation—among Chinese adolescents. The analytic sample comprised 802 senior high school students (*M*_age_ = 15.72 years, *SD* = 0.89; 57.0% female) who completed measures of PIU and FTP twice at a six-month interval. An observed-variable cross-lagged panel model was estimated while accounting for autoregressive stability, prospective associations among FTP dimensions, and effects of gender, age, and baseline self-reported academic standing. At both waves, PIU was negatively correlated with future-positive and positively correlated with future-negative and task orientation. Higher PIU at Time 1 was prospectively associated with lower future-positive and higher future-negative at Time 2. In the reverse direction, higher task orientation at Time 1 was prospectively associated with higher PIU at Time 2. No paired cross-lagged coefficients differed significantly in magnitude. The findings indicate modest, dimension-specific longitudinal associations between PIU and FTP. Distinguishing among FTP dimensions may improve theoretical understanding and inform broader school-based assessment and support.

## 1. Introduction

Internet use has become nearly universal among Chinese minors. According to the *Sixth Survey Report on Internet Use among Minors in China*, a nationwide survey covering primary- and secondary-school students across all 31 provincial-level regions of mainland China and the Xinjiang Production and Construction Corps, China, had approximately 196 million minor Internet users in 2023, representing an Internet penetration rate of 97.3% (Central Committee of the Communist Youth League of China & China Internet Network Information Center, 2024).

Against this backdrop, difficulties in regulating online behavior have become an important concern. Problematic Internet use (PIU) refers to a generalized pattern of persistent difficulty controlling Internet use accompanied by distress or impairment in academic, interpersonal, or everyday functioning (Fineberg et al., 2022). Higher levels of PIU have been associated with poorer academic functioning, lower sleep quality, health problems, and aggressive behavior (Hà et al., 2023; Leung & Lee, 2012; Zhao et al., 2022; Zhou et al., 2022). Accordingly, it is important to identify psychological factors prospectively associated with PIU.

One potentially relevant factor is time perspective (Gu et al., 2026; Miceli et al., 2022; Shen et al., 2026). Time perspective refers to the habitual process through which individuals organize personal and social experiences within past, present, and future temporal frames, thereby shaping judgments, decisions, and behavior (Zimbardo & Boyd, 1999). Future-oriented representations become increasingly important during adolescence, as educational, occupational, and broader developmental goals require anticipation, evaluation, and planning (Hao et al., 2024a, 2024b). Future time perspective (FTP) refers to how individuals anticipate, evaluate, and organize their personal future (Kooij et al., 2018; Lyu & Huang, 2016).

Associations between FTP and PIU or related problematic digital behaviors have been reported among South Korean and Chinese university students (Kim et al., 2017; Y. Liu et al., 2023; Mao et al., 2026). However, whether these findings generalize to Chinese secondary-school adolescents remains uncertain. In addition, these studies have not consistently distinguished the three FTP dimensions examined in the present study, potentially obscuring dimension-specific patterns. The present study therefore distinguished among future-positive, future-negative, and task orientation (Li, 2022). Future-positive and future-negative reflect favorable and unfavorable evaluations of the future, respectively (Lyu & Huang, 2016), whereas task orientation describes a pattern in which life is perceived as being dominated by tasks, with insufficient time available for family, friends, or leisure (Li, 2022). Examining these dimensions separately may clarify whether PIU shows different prospective associations with later future-positive, future-negative, and task orientation, and whether each dimension is prospectively associated with later PIU.

### 1.1. Prospective Associations from Problematic Internet Use to Future Time Perspective

A plausible mechanism linking PIU with later FTP is the displacement of offline experiences. Early research proposed that extensive online engagement may displace offline social involvement (Kraut et al., 1998). Recent longitudinal findings provide indirect support for related processes. Among rural Chinese adolescents, longer Internet use was associated with poorer mental health partly through shorter sleep duration and reduced parent–adolescent communication (Ma & Sheng, 2023). Similarly, adolescents’ compulsive Internet use was prospectively associated with small declines in perceived social support across four years (Donald et al., 2024). Together, these findings suggest that poorly regulated Internet involvement may reduce the availability or quality of experiences and social resources that inform adolescents’ evaluations of their future.

This pathway may be particularly relevant to the future-positive and future-negative dimensions. In a four-year longitudinal study, compulsive Internet use was prospectively associated with reductions in adolescents’ hope (Donald et al., 2019). Although hope is conceptually distinct from future-positive, both involve favorable expectations about the future. Moreover, because representations of future events are partly constructed from current and remembered experiences (Schacter et al., 2017), academic, emotional, and interpersonal difficulties associated with PIU may provide less favorable material for imagining one’s future. Higher PIU may therefore be prospectively associated with lower future-positive and higher future-negative.

A related but distinct mechanism may apply to task orientation. Poorly regulated Internet use may displace time that would otherwise be available for study, rest, family, friends, or leisure. Because academic and other daily obligations remain, substantial time spent online may compress the time available for completing these tasks. Adolescents may consequently experience greater time pressure and a stronger sense that their lives are dominated by tasks, both of which are central features of task orientation. PIU may therefore be prospectively associated with higher subsequent task orientation, although direct evidence for this pathway remains scarce.

### 1.2. Prospective Associations from Future Time Perspective to Problematic Internet Use

Another possible temporal direction concerns whether FTP is prospectively associated with PIU. The Interaction of Person–Affect–Cognition–Execution (I-PACE) model provides a framework for understanding how person-related characteristics interact with affective and cognitive responses and executive-control processes in the development of poorly regulated online behavior (Brand et al., 2019). Within this framework, FTP may shape the salience of long-term goals and the regulation of immediate online rewards. Future-positive may increase attention to longer-term consequences (Hao et al., 2024a, 2024b), whereas future-negative may heighten distress and uncertainty, making online distraction or emotional compensation more appealing (Brand et al., 2019; Kardefelt-Winther, 2014).

Existing findings provide indirect support for these possibilities. Among Chinese adolescents, positive future expectations were negatively associated with PIU (Du & Lyu, 2021). FTP was also found to strengthen the association between higher self-control and lower mobile phone addiction tendencies among Chinese adolescents (Peng et al., 2022), while positive-orientation indicators such as optimism have been associated with lower PIU (Hinić et al., 2020). However, this evidence is predominantly cross-sectional or based on constructs adjacent to future-positive and future-negative. Whether these two dimensions are prospectively associated with later PIU remains unclear.

Task orientation may operate through a different process. In the present study, it refers to a tendency to perceive life as dominated by tasks and to feel that insufficient time remains for family, friends, or leisure (Li, 2022). Educational applications and online learning platforms are used for online homework and instruction among secondary-school students (L. Chen et al., 2024; Xu & Deng, 2024). This digitally mediated learning context raises the possibility that adolescents with higher task orientation may rely more heavily on Internet-based resources to manage academic demands. Such task-related reliance does not itself constitute PIU, but it may increase opportunities for instrumental Internet use to extend into poorly regulated use. Whether task orientation is prospectively associated with later PIU remains unclear because direct evidence is scarce.

Taken together, the preceding theoretical accounts suggest that PIU and FTP may be dynamically related. Future-oriented evaluations may shape motives for and control over Internet use, whereas repeated poorly regulated Internet use may alter the academic, interpersonal, and emotional experiences from which future evaluations are constructed.

### 1.3. The Present Study

To address the limited evidence concerning temporal ordering and dimension-specific associations, the present study used a two-wave longitudinal design to examine the prospective associations between PIU and future-positive, future-negative, and task orientation among Chinese senior high school students.

The model accounted for autoregressive stability, prospective associations among the FTP dimensions, and the specified covariates. We also compared the paired cross-lagged coefficients to determine whether the two prospective directions differed significantly in magnitude. Accordingly, the study addressed the following research questions:

RQ1: Is PIU prospectively associated with subsequent future-positive, future-negative, and task orientation among Chinese adolescents?

RQ2: Are future-positive, future-negative, and task orientation prospectively associated with subsequent PIU among Chinese adolescents?

RQ3: Do the paired cross-lagged coefficients between PIU and each FTP dimension differ significantly in magnitude?

## 2. Methods

### 2.1. Participants and Procedure

A convenience sampling approach was used to recruit students from one senior high school in Henan Province, China. The school was selected on the basis of accessibility and willingness to cooperate. Within the school, all intact classes in the participating grades were included, and all students in these classes were invited to participate.

After permission was obtained from the school administration, trained graduate students in psychology visited the school to conduct both waves of data collection. Students completed an electronic questionnaire using computers in the school computer laboratories. Before each administration, the research assistants provided standardized instructions, explained the voluntary and confidential nature of participation, and answered questions concerning the survey procedure. Students then completed the questionnaire independently.

Data collection was organized by class. A total of 814 students participated in the first survey (Time 1; T1) in November 2021. The second survey (Time 2; T2) was conducted six months later using the same procedure. Students who were temporarily absent because of illness, approved leave, or other reasons were invited to complete the questionnaire during supplementary administration after confirming that they remained willing to participate. Through the regular and supplementary administrations, responses from all 814 baseline participants were collected at T2 and matched across the two waves.

Responses from T1 and T2 were matched using the combination of each student’s name and identification number. Identifying information was stored separately from survey responses and was removed after longitudinal matching. The electronic questionnaire required a response to every item before submission; therefore, submitted questionnaires contained no item-level missing responses. During data-quality screening, 12 participants were excluded because they selected the same response option for all 12 FTP items at one or both waves. No additional cases were excluded. The final analytic sample comprised 802 participants with complete and usable data at both waves, and no missing-data imputation was performed.

Among the 802 participants included in the analyses, the mean age was 15.72 years (SD = 0.89), and 57.0% were female. In addition, 46 participants (5.7%) reported being only children, and 618 (77.1%) lived in rural areas.

The study was conducted in accordance with the Declaration of Helsinki. The longitudinal data collection, which began in November 2021 and included a follow-up survey six months later, was conducted under the overarching project approval granted by the Ethics Committee of the Faculty of Psychology, Southwest University, before data collection began (protocol code H21081; approved on 25 May 2021). The subsequent manuscript-specific analysis of the data was additionally covered by a related sub-project approval granted by the same ethics committee (protocol code H22103; approved on 20 October 2022). Written informed consent was obtained from the legal guardians of all participants before data collection. Students were informed that participation was voluntary, that their responses would be kept confidential, and that they could withdraw from the study at any time without penalty.

### 2.2. Measures

#### 2.2.1. Problematic Internet Use

PIU was assessed using the 10-item Internet Addiction Scale (Shek et al., 2008), which has continued to be used in recent studies of Chinese student samples (Huang et al., 2026; K. Liu et al., 2025; Yan et al., 2023). The 10 items are rated dichotomously (0 = no, 1 = yes) and assess tolerance, impaired control, withdrawal-like discomfort, use of the Internet to regulate negative mood, concealment of excessive involvement, functional interference, and remaining online longer than intended.

For the longitudinal analyses, item responses were averaged to produce scores ranging from 0 to 1, with higher scores indicating higher levels of PIU. According to previous studies (Huang et al., 2026; Shek et al., 2008), participants endorsing four or more items were classified as screening positive.

Given the dichotomous response format, internal consistency was evaluated using the Kuder–Richardson Formula 20 (KR-20) coefficient and corrected item–total correlations. The KR-20 coefficients were 0.806 at T1 and 0.855 at T2. Corrected item–total correlations ranged from 0.352 to 0.574 at T1 and from 0.354 to 0.647 at T2.

A categorical one-factor confirmatory factor analysis was conducted separately at each wave using the estimation procedure described in Section 2.3. At T1, the model yielded χ^2^(35) = 151.39, CFI = 0.976, TLI = 0.970, RMSEA = 0.064, and SRMR = 0.089, with standardized factor loadings ranging from 0.626 to 0.846. At T2, the model yielded χ^2^(35) = 57.05, CFI = 0.997, TLI = 0.997, RMSEA = 0.028, and SRMR = 0.051, with standardized loadings ranging from 0.603 to 0.880.

#### 2.2.2. Future Time Perspective

FTP was assessed using the future dimension of the Balanced Time Perspective Scale (Li, 2022). The measure contains 12 items rated on a 5-point scale ranging from 1 (strongly disagree) to 5 (strongly agree) and comprises three four-item dimensions: future-positive (e.g., “Anticipating my future life fills me with hope”), future-negative (e.g., “I feel that my future is hopeless”), and task orientation (e.g., “I never feel like I have enough time”). Item scores were averaged within each dimension, with higher scores indicating higher levels of that dimension.

For future-positive, Cronbach’s α coefficients were 0.793 at T1 and 0.873 at T2, and ordinal ω coefficients were 0.861 and 0.913, respectively. For future-negative, α coefficients were 0.863 and 0.890, and ordinal ω coefficients were 0.901 and 0.922. For task orientation, α coefficients were 0.678 and 0.758, and ordinal ω coefficients were 0.740 and 0.799.

The hypothesized three-factor model showed good fit at both waves: T1, χ^2^(51) = 156.77, CFI = 0.974, TLI = 0.967, RMSEA = 0.051, and SRMR = 0.048; T2, χ^2^(51) = 200.03, CFI = 0.975, TLI = 0.967, RMSEA = 0.060, and SRMR = 0.045.

#### 2.2.3. Baseline Self-Reported Academic Standing

Baseline self-reported academic standing was assessed at T1 using a single item: “How would you rate your academic performance this semester?” Responses were scored on a five-point scale (1 = low, 2 = lower-middle, 3 = middle, 4 = upper-middle, and 5 = high), with higher scores indicating better self-rated academic performance.

### 2.3. Data Analysis

All analyses were based on the final analytic sample of 802 participants. Data preparation, descriptive statistics, reliability analyses, Pearson correlation analyses, wave-specific FTP confirmatory factor analyses, and longitudinal measurement-invariance analyses were conducted in Python Version 3.10 using the pandas, NumPy, and SciPy packages. The categorical confirmatory factor analyses for the dichotomous PIU items and the observed-variable cross-lagged panel model were estimated in Mplus Version 8.3. For the dichotomous problematic Internet use items, categorical confirmatory factor analyses were conducted using the weighted least squares mean- and variance-adjusted estimator (WLSMV), a robust diagonally weighted least-squares estimator based on tetrachoric correlations, with theta parameterization and all 10 items specified as categorical. The wave-specific FTP confirmatory factor analyses and longitudinal measurement-invariance models were estimated using ordinary maximum likelihood estimation, with the 5-point items treated as continuous indicators. Internal consistency for the dichotomously scored PIU items was evaluated using the KR-20 coefficient and corrected item–total correlations. For the 5-point FTP items, Cronbach’s α and ordinal ω were calculated.

Longitudinal measurement invariance was evaluated sequentially at the configural, metric, scalar, and strict levels. A decrease in CFI of less than 0.010 between successive models was used as the primary criterion for supporting invariance (Cheung & Rensvold, 2002). For FTP, the longitudinal invariance models were evaluated using the three-factor measurement structure. For PIU, longitudinal invariance models treating the dichotomous indicators as continuous were examined as sensitivity analyses.

Harman’s single-factor test was conducted as a preliminary diagnostic of potential common-method variance. Means, standard deviations, screening proportions, and zero-order associations among the wave-specific composite scores were then calculated. Pearson product–moment correlations were used to examine concurrent associations and cross-wave rank-order stability. Each correlation was calculated across participants using one composite score for each construct at each wave.

To examine the longitudinal associations between PIU and the three dimensions of FTP, an observed-variable cross-lagged panel model was estimated using ordinary maximum likelihood estimation. Composite scores for PIU, future-positive, future-negative, task orientation, age, and baseline self-reported academic standing were standardized before analysis. Gender was coded as 0 = female and 1 = male.

All four T1 focal variables were entered simultaneously as predictors of all four T2 variables, thereby estimating four autoregressive paths, six focal cross-lagged paths between PIU and the FTP dimensions, and six prospective paths among the FTP dimensions. All four T1 focal variables were allowed to covary, and the residuals of the four T2 outcomes were also allowed to covary.

Covariate paths were prespecified based on prior evidence. Gender was included as a predictor of T2 PIU (Mo et al., 2020), age was included as a predictor of the three T2 FTP dimensions (Li et al., 2023), and baseline self-reported academic standing was included as a predictor of all four T2 outcomes (Hao et al., 2024a). Gender, age, and baseline self-reported academic standing were allowed to covary with one another and with all T1 focal variables.

Model fit was evaluated using the chi-square statistic, CFI, TLI, RMSEA, and SRMR. Focal paths were evaluated primarily using 95% bias-corrected bootstrap confidence intervals based on 5000 case-resampling bootstrap samples. *p* values were also reported.

Finally, the paired cross-lagged paths between PIU and each FTP dimension were formally compared. A directional difference was considered supported when the 95% bootstrap confidence interval for the difference estimate did not include zero.

## 3. Results

Harman’s single-factor test indicated that the first factor explained 23.00% of the total variance at T1 and 27.98% at T2.

### 3.1. Longitudinal Measurement Invariance

Longitudinal measurement-invariance analyses were conducted for PIU and FTP. For both constructs, changes in CFI were smaller than 0.010 at each successive step. Thus, the results (see Table 1) supported strict longitudinal invariance.

### 3.2. Descriptive Statistics

Descriptive statistics and Pearson product–moment correlations are presented in Table 2. The cross-wave correlations between the same constructs were positive. At both waves, PIU was negatively correlated with future-positive and positively correlated with future-negative and task orientation.

Applying the original screening criterion of four or more affirmative responses (Huang et al., 2026; Shek et al., 2008), 270 participants (33.7%) screened positive for elevated PIU at T1, and 323 participants (40.3%) screened positive at T2. A total of 167 participants (20.8%) met the screening threshold at both waves.

### 3.3. Cross-Lagged Associations Between Problematic Internet Use and Future Time Perspective

An observed-variable cross-lagged panel model was estimated to examine the prospective associations between PIU and the three dimensions of FTP. The model yielded generally favorable fit indices, although the TLI was marginally below 0.90: χ^2^(4) = 14.88, CFI = 0.992, TLI = 0.897, RMSEA = 0.058, and SRMR = 0.016.

All autoregressive paths were positive and statistically significant (see Table 3). PIU showed the highest cross-wave stability (β = 0.423, *p* < 0.001, 95% CI [0.345, 0.498]), followed by future-positive (β = 0.289, *p* < 0.001, 95% CI [0.210, 0.366]), future-negative (β = 0.264, *p* < 0.001, 95% CI [0.179, 0.344]), and task orientation (β = 0.230, *p* < 0.001, 95% CI [0.156, 0.301]).

After accounting for autoregressive stability, prospective associations among the FTP dimensions, and the theoretically specified covariates, higher PIU at T1 was prospectively associated with lower future-positive at T2 (β = −0.072, *p* = 0.029, 95% CI [−0.147, −0.002]) and higher future-negative at T2 (β = 0.119, *p* < 0.001, 95% CI [0.050, 0.188]). PIU at T1 also showed a small positive prospective association with task orientation at T2 (β = 0.070, *p* = 0.044), but the 95% bias-corrected bootstrap confidence interval included zero [−0.001, 0.138]; this path was therefore not interpreted as supported.

In the reverse direction, higher task orientation at T1 was prospectively associated with higher PIU at T2 (β = 0.075, *p* = 0.033, 95% CI [0.003, 0.143]). Future-positive (*p* = 0.951) and future-negative (*p* = 0.213) showed no prospective associations with subsequent PIU.

The paired path comparisons were not significant for future-positive (*p* = 0.168), future-negative (*p* = 0.243), or task orientation (*p* = 0.929). Thus, none of the paired prospective effects differed significantly in magnitude.

## 4. Discussion

This study used a two-wave cross-lagged design to examine dimension-specific prospective associations between PIU and FTP among Chinese senior high school students. After accounting for baseline stability and prospective associations among the FTP dimensions and the specified covariates, higher levels of PIU at T1 were prospectively associated with lower future-positive and higher future-negative at T2. In the reverse direction, higher task orientation at T1 was prospectively associated with higher PIU at T2, whereas future-positive and future-negative showed no prospective associations with subsequent PIU. The PIU-to-task orientation path was not supported by the bias-corrected bootstrap confidence interval, and none of the paired cross-lagged coefficients differed significantly in magnitude. Before considering these dimension-specific findings, the temporal stability and magnitude of the observed associations warrant closer attention.

### 4.1. Magnitude and Temporal Stability of the Associations

The same-construct correlations across the six-month interval ranged from 0.28 to 0.44, indicating partial rather than strong rank-order stability. This pattern may partly reflect developmental change during adolescence. A five-wave longitudinal study found that overall FTP increased across adolescence and that age was positively associated with its initial level (Li et al., 2023). Although mean-level growth does not imply lower rank-order stability itself, individual differences in developmental trajectories may alter adolescents’ relative standing over time. Contextual changes during the COVID-19 period may also have contributed to temporal variation. Changes in school arrangements and differences in exposure to online learning may have affected both adolescents’ Internet-use patterns and their evaluations of the future. Related longitudinal work has documented changes in adolescents’ family and academic functioning across pandemic and post-pandemic schooling contexts (Wen et al., 2026), underscoring the potential importance of historical context.

The concurrent correlations between PIU and the FTP dimensions were small (|r| = 0.17–0.22), corresponding to approximately 3–5% shared variance at the observed-score level. This limited overlap is consistent with the conceptual distinction between difficulties regulating Internet use and evaluations of the personal future. The association between these constructs may also be partly indirect, operating through more proximal processes such as self-control (Kim et al., 2017), coping motives (Brand et al., 2019; Kardefelt-Winther, 2014), and maladaptive Internet-related cognitions (Davis, 2001; Mai et al., 2012). In addition, the use of a global PIU score may obscure activity-specific patterns, because different forms of online activity may show different associations with future-positive, future-negative, and task orientation.

The supported cross-lagged effects were also modest in absolute magnitude (|β| = 0.072–0.119). Relative to the distribution-based benchmarks proposed by Orth et al. (2024), the PIU-to-future-positive and task orientation-to-PIU effects were close to the medium benchmark, whereas the PIU-to-future-negative effect approached the large benchmark. These relative benchmarks do not, however, imply substantial absolute predictive effects: the confidence intervals for the PIU-to-future-positive and task-orientation-to-PIU paths extended very close to zero.

### 4.2. Future-Positive, Future-Negative, and Problematic Internet Use

Higher levels of PIU at T1 were prospectively associated with lower future-positive and higher future-negative at T2. This pattern is consistent with longitudinal evidence showing that compulsive Internet use preceded subsequent reductions in adolescents’ hope and, to a lesser extent, self-esteem (Donald et al., 2019). The present findings add to this literature by indicating that PIU was prospectively associated not only with weaker positive evaluations of the future but also with stronger negative future evaluations.

One possible explanation involves the displacement of developmentally important offline experiences. PIU may consume time and attentional resources that would otherwise support adequate sleep, family communication, academic engagement, and supportive relationships (Donald et al., 2024; Ma & Sheng, 2023). These domains provide experiential material which adolescents use to evaluate and imagine their personal future (Schacter et al., 2017).

Another possible explanation concerns changes in the affective functions of Internet use as problematic involvement develops. The I-PACE model distinguishes gratification obtained from online activities from compensation for unmet needs or negative emotional states and suggests that the relative roles of these reinforcement processes may change across stages of addictive behavior (Brand et al., 2019, 2025; Wegmann et al., 2022). Internet use may initially provide pleasure, stimulation, or temporary relief; however, repeated poorly regulated use may become increasingly associated with habitual engagement, craving-like experiences, and relief from negative affect rather than a sustained positive experience.

In the reverse direction, neither future-positive nor future-negative was prospectively associated with PIU six months later. This finding contrasts with the concurrent associations observed at both waves and with previous studies linking positive future expectations or broader positive orientation to lower PIU (Du & Lyu, 2021; Hinić et al., 2020). However, these studies were primarily cross-sectional or examined conditional associations involving constructs adjacent to future-positive and future-negative. They therefore support the presence of concurrent or indirect relationships but do not establish that these future evaluations are uniquely prospectively associated with later PIU.

Future evaluations may function as relatively distal factors with links to PIU that emerge through more proximal affective, cognitive, and self-regulatory processes. Cognitive-behavioral accounts distinguish broad psychosocial vulnerabilities from Internet-related cognitions that may more directly sustain poorly regulated online behavior (Davis, 2001; Mai et al., 2012). Extending this perspective, the I-PACE model highlights coping motives, affective responses, Internet-related cognitions, and executive-control processes in the development and maintenance of poorly regulated Internet use (Brand et al., 2019). Consistent with this possibility, intolerance of uncertainty partially accounted for the cross-sectional association between future expectations and PIU among Chinese adolescents (Du & Lyu, 2021).

### 4.3. Task Orientation and Problematic Internet Use

Higher task orientation at T1 showed a small prospective association with higher PIU at T2. In the present study, task orientation refers to a tendency to perceive life as dominated by tasks and to experience insufficient time for family, friends, or leisure (Li, 2022). This dimension therefore reflects not simply the amount of schoolwork adolescents complete, but their subjective experience of persistent task demands and limited discretionary time.

One possible explanation concerns the increasingly digitalized context of adolescent learning. Educational applications and online platforms are used for homework, information seeking, instruction, and communication among secondary-school students (L. Chen et al., 2024; Xu & Deng, 2024). In this context, adolescents who experience persistent task demands may perceive Internet-based resources as increasingly necessary for managing academic obligations. Although academic stress is conceptually distinct from task orientation, related evidence supports a broader connection between sustained academic demands and poorly regulated Internet use. A three-level meta-analysis found a positive association between academic stress and PIU across adolescent samples (Z. Chen et al., 2025). In a three-wave study, academic stress and PIU showed reciprocal positive within-person associations (Z. Chen & Guo, 2026). A separate longitudinal study found that within-person increases in academic stress were associated with higher subsequent PIU among late adolescents (Wang et al., 2026).

The I-PACE model provides a framework for understanding how this association might develop. Task-related Internet use is initially goal-directed and does not constitute PIU. However, persistent task pressure may increase both the perceived necessity of Internet use and the appeal of immediately rewarding online activities. Coping motives, affective responses, Internet-related cognitions, and self-regulatory capacity may then influence whether Internet use remains instrumental or becomes increasingly difficult to control (Brand et al., 2019; Kardefelt-Winther, 2014). Adolescents may, for example, move between completing academic tasks and using online activities to obtain temporary relief from stress or fatigue. Repeated reliance on the same digital environment for both task completion and affect regulation may make disengagement more difficult.

### 4.4. Practical Implications

The findings may inform school-based assessment and support. When adolescents report persistent difficulty controlling their Internet use, educators and school counselors could also assess how they evaluate their future and whether they perceive their lives as dominated by tasks.

The association between task orientation and subsequent PIU also highlights the importance of distinguishing necessary task-related Internet use from poorly regulated use. Schools and families could support clearer boundaries among online study, entertainment, rest, and offline activities, particularly for students who experience persistent task pressure or difficulty disengaging from online activities.

### 4.5. Limitations

Several limitations should be considered. First, participants were recruited through convenience sampling from one senior high school in Henan Province, and most lived in rural areas. The findings may therefore not generalize to adolescents from other regions, school types, or demographic backgrounds. Replication using multi-site and more diverse samples is needed.

Second, T1 data were collected in November 2021 and T2 data six months later, during the COVID-19 period and shortly after the introduction of stricter national restrictions on the provision of online gaming services to minors in China (National Press and Publication Administration, 2021). Changes in schooling, social interaction, and patterns of Internet use may therefore have shaped both PIU and FTP. Because these contextual influences were not directly assessed, replication across different historical and regulatory periods is needed.

Third, the traditional two-wave cross-lagged panel model has important interpretive limitations. It does not distinguish stable between-person differences from within-person deviations over time (Hamaker et al., 2015). Because only two measurement occasions were available, a random-intercept cross-lagged panel model could not be estimated. The findings indicate prospective associations conditional on baseline levels but do not establish within-person processes or causal effects. Future research should include at least three waves and use random-intercept cross-lagged models, latent growth models, or intensive longitudinal designs to separate within-person change from stable between-person differences.

Fourth, although the model included gender, age, and baseline self-reported academic standing, other family, emotional, self-regulatory, and Internet-use factors were not assessed. In particular, prior Internet-use experience and average daily Internet-use time were not assessed and therefore could not be reported descriptively or included as covariates. Their omission limits our ability to distinguish associations involving problematic regulation from those involving overall Internet exposure. Future research should incorporate a broader range of psychosocial factors and more detailed assessments of Internet-use patterns.

Fifth, the PIU measure provided a global assessment of poorly regulated Internet use and did not distinguish among specific online activities. Different forms of Internet use, such as social media, short-form video, gaming, and online learning, may have different associations with FTP. In addition, because the measure was developed in an earlier Internet environment, some items may not fully reflect contemporary mobile and platform-based patterns of use. The comparatively modest reliability of task orientation at T1 may also have reduced the precision of paths involving this dimension. Future studies should use updated, activity-sensitive PIU measures and more reliable assessments of task orientation.

Sixth, the present study focused only on FTP and did not assess past- or present-oriented dimensions of time perspective. In particular, present-hedonistic orientation may be relevant to poorly regulated Internet use because it emphasizes immediate reward and gratification. Future research should examine PIU within a more comprehensive time-perspective framework that simultaneously considers past, present, and future orientations.

Finally, all focal variables were assessed using adolescent self-report measures. The observed associations may therefore have been influenced by shared method variance, recall limitations, or socially desirable responding. Future research should incorporate multi-informant assessments and objective indicators of digital behavior.

## 5. Conclusions

This two-wave longitudinal study identified dimension-specific prospective associations between PIU and FTP among senior high school students in Henan Province, China. Higher levels of PIU were prospectively associated with less positive and more negative future evaluations six months later, whereas task orientation showed a small prospective association with subsequent PIU. Future-positive and future-negative were not prospectively associated with subsequent PIU, and the bias-corrected bootstrap confidence interval did not support the PIU-to-task orientation path. Moreover, none of the paired cross-lagged coefficients differed significantly in magnitude.

Theoretically, these dimension-specific findings highlight the importance of distinguishing future-positive, future-negative, and task orientation rather than treating FTP as a single global construct. Practically, the findings suggest that school-based assessment may consider difficulties in regulating Internet use together with adolescents’ future evaluations and perceived task pressure, while support for highly task-oriented students may emphasize clearer boundaries among online study, recreational use, rest, and offline activities. Because the traditional two-wave cross-lagged model does not separate stable between-person differences from within-person change, the results concern prospective associations rather than causal or within-person processes.

## Figures and Tables

**Table 1:** Longitudinal measurement invariance of all variables.

Construct and Model	*χ*^2^(*df*)	CFI	TLI	RMSEA	SRMR	Δ*χ*^2^(Δ*df*)	*p*	ΔCFI
problematic Internet use								
M1 Configural	511.14 (159)	0.924	0.909	0.053	0.044			
M2 Metric	520.84 (168)	0.924	0.914	0.051	0.046	9.71 (9)	0.375	<0.001
M3 Scalar	557.88 (178)	0.918	0.913	0.052	0.046	37.03 (10)	<0.001	0.006
M4 Strict	597.01 (188)	0.912	0.911	0.052	0.047	39.13 (10)	<0.001	0.006
future time perspective								
M1 Configural	515.90 (225)	0.972	0.966	0.040	0.042			
M2 Metric	539.20 (234)	0.971	0.966	0.040	0.043	23.30 (9)	0.006	0.001
M3 Scalar	615.78 (246)	0.965	0.960	0.043	0.043	76.58 (12)	<0.001	0.006
M4 Strict	681.52 (258)	0.960	0.957	0.045	0.045	65.74 (12)	<0.001	0.005

Note. M1 = configural invariance; M2 = metric invariance; M3 = scalar invariance; M4 = strict invariance.

**Table 2:** Bivariate correlations and descriptive statistics.

Variable	*M*	*SD*	1	2	3	4	5	6	7	8
1. PIU T1	0.28	0.26	—							
2. FP T1	3.81	0.75	−0.17 **	—						
3. FN T1	2.16	0.87	0.18 **	−0.45 **	—					
4. TO T1	2.88	0.76	0.17 **	−0.11 **	0.42 **	—				
5. PIU T2	0.32	0.30	0.44 **	−0.09 **	0.15 **	0.16 **	—			
6. FP T2	3.80	0.86	−0.16 **	0.38 **	−0.32 **	−0.16 **	−0.21 **	—		
7. FN T2	2.27	0.95	0.20 **	−0.32 **	0.39 **	0.21 **	0.22 **	−0.47 **	—	
8. TO T2	2.74	0.83	0.14 **	−0.12 **	0.22 **	0.28 **	0.20 **	−0.19 **	0.55 **	—

Note. PIU = problematic Internet use; FP = future-positive; FN = future-negative; TO = task orientation; T1 = Time 1; T2 = Time 2. ** *p* < 0.01.

**Table 3:** Standardized autoregressive and cross-lagged coefficients.

Path	β	*p*	95% CI
autoregressive effects			
PIU T1 → PIU T2	0.423	<0.001	[0.345, 0.498]
FP T1 → FP T2	0.289	<0.001	[0.210, 0.366]
FN T1 → FN T2	0.264	<0.001	[0.179, 0.344]
TO T1 → TO T2	0.230	<0.001	[0.156, 0.301]
problematic Internet use → future time perspective			
PIU T1 → FP T2	−0.072	0.029	[−0.147, −0.002]
PIU T1 → FN T2	0.119	<0.001	[0.050, 0.188]
PIU T1 → TO T2	0.070	0.044	[−0.001, 0.138]
future time perspective → problematic Internet use			
FP T1 → PIU T2	0.002	0.951	[−0.077, 0.072]
FN T1 → PIU T2	0.049	0.213	[−0.034, 0.138]
TO T1 → PIU T2	0.075	0.033	[0.003, 0.143]
cross-dimensional FTP effects			
FN T1 → FP T2	−0.151	<0.001	[−0.228, −0.067]
TO T1 → FP T2	−0.056	0.117	[−0.129, 0.013]
FP T1 → FN T2	−0.164	<0.001	[−0.242, −0.089]
TO T1 → FN T2	0.057	0.106	[−0.012, 0.130]
FP T1 → TO T2	−0.051	0.189	[−0.125, 0.022]
FN T1 → TO T2	0.093	0.024	[0.013, 0.179]

Note. PIU = problematic Internet use; FP = future-positive; FN = future-negative; TO = task orientation; T1 = Time 1; T2 = Time 2.

## Data Availability

The data that support the findings of this study are available from the corresponding author upon reasonable request. The data are not publicly available due to privacy and ethical restrictions.
